# Six-Dimensional Pose Estimation of Molecular Sieve Drying Package Based on Red Green Blue–Depth Camera

**DOI:** 10.3390/s25020462

**Published:** 2025-01-15

**Authors:** Yibing Chen, Songxiao Cao, Qixuan Wang, Zhipeng Xu, Tao Song, Qing Jiang

**Affiliations:** College of Metrology Measurement and Instrument, China Jiliang University, Hangzhou 310018, China; p22020854011@cjlu.edu.cn (Y.C.); p24020854104@cjlu.edu.cn (Q.W.); xuzhipeng@cjlu.edu.cn (Z.X.); songtao@cjlu.edu.cn (T.S.); 06a0203051@cjlu.edu.cn (Q.J.)

**Keywords:** three-dimensional object recognition, RGB-D camera, visual guidance, six-dimensional pose estimation

## Abstract

This paper aims to address the challenge of precise robotic grasping of molecular sieve drying bags during automated packaging by proposing a six-dimensional (6D) pose estimation method based on an red green blue-depth (RGB-D) camera. The method consists of three components: point cloud pre-segmentation, target extraction, and pose estimation. A minimum bounding box-based pre-segmentation method was designed to minimize the impact of packaging wrinkles and skirt curling. Orientation filtering combined with Euclidean clustering and Principal Component Analysis (PCA)-based iterative segmentation was employed to accurately extract the target body. Lastly, a multi-target feature fusion method was applied for pose estimation to compute an accurate grasping pose. To validate the effectiveness of the proposed method, 102 sets of experiments were conducted and compared with classical methods such as Fast Point Feature Histograms (FPFH) and Point Pair Features (PPF). The results showed that the proposed method achieved a recognition rate of 99.02%, processing time of 2 s, pose error rate of 1.31%, and spatial position error of 3.278 mm, significantly outperforming the comparative methods. These findings demonstrated the effectiveness of the method in addressing the issue of accurate 6D pose estimation of molecular sieve drying bags, with potential for future applications to other complex-shaped objects.

## 1. Introduction

**Figure 1:**
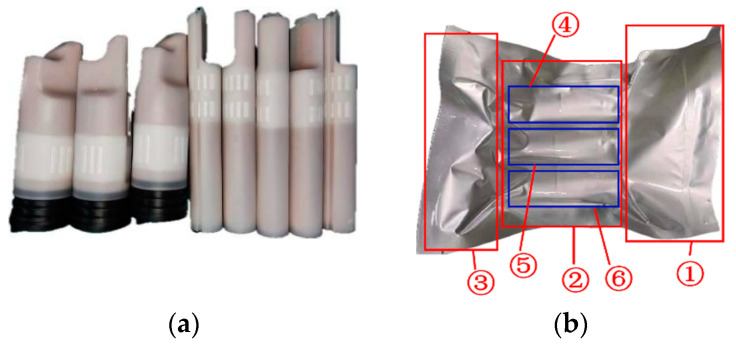
Molecular sieve drying pack. (**a**) NMSDP before vacuuming; (**b**) NMSDP after vacuuming; ① front section; ② middle section; ③ rear section; ④ target entity 1; ⑤ target entity 2; ⑥ target entity 3.

In the contemporary automotive industry, there is a growing demand for lightweight materials and advanced manufacturing technologies in the design of automotive refrigeration systems. This trend is driven by the need to reduce vehicle weight while enhancing refrigeration efficiency and performance. As depicted in Figure 1a, the industry has adopted a novel design for the traditional receiver drier, namely a molecular sieve dryer pack (NMSDP). This innovation features a detachable filtering structure, ensuring optimal filtration during operation. This design eliminates the need to replace the receiver drier, thereby reducing operational costs, enhancing utilization efficiency, and extending the service life of the receiver drier.

As illustrated in Figure 1, while some manufacturers possess the capacity to automate the production of NMSDP products, the subsequent packaging process remains a manual task. To ensure consistent packaging quality and to alleviate workers from the effects of monotonous and repetitive tasks, it is necessary to achieve full-line, end-to-end automated production. Consequently, an automated packaging system suitable for NMSDP has been designed. During the production process, the dryer pack was vacuum-sealed and then fed by a robot. The vacuum-sealed packaging had a complex surface structure and lacked a fixed posture, making it difficult for traditional robotic automation systems to perform precise localization and grasping. This paper addresses the issue of insufficient positioning and grasping accuracy of NMSDP products by robots in the automated packaging system.

Three-dimensional (3D) object recognition technology [1] plays a pivotal role in industrial automation and robotic vision. It enables machines to recognize and locate target objects in complex environments. Three-dimensional point clouds [2], as a form of data expressing the geometric shape of objects, are acquired through various methods, such as stereo cameras [3], light detection and ranging (LiDAR) [4], and red green blue-depth (RGB-D) cameras [5]. Each method has its own strengths and limitations; for example, LiDAR is particularly effective in outdoor environments, whereas RGB-D cameras are commonly used in indoor scenarios. Additionally, the acquisition of three-dimensional point cloud data is not affected by environmental lighting, thereby avoiding issues with lighting and pose encountered in two-dimensional images. The predominant methodologies for three-dimensional object recognition include the following: local features-based recognition [6], global features-based recognition [7], point cloud-based approaches [8], and machine learning-based methods [2].

Methods based on local features focus on identifying local geometric characteristics of objects, such as corners and edges. They typically exhibit robustness against noise and occlusion but are computationally intensive. Rusu R B et al. [8]. proposed Point Feature Histograms (PFH) and Fast Point Feature Histogram (FPFH), which construct feature descriptors by statistically analyzing the angles between neighboring points. The significance of local features in three-dimensional object recognition has also been emphasized by Guo et al. [9].

Methods based on global features seek to capture the overall shape and structure of objects. These methods rely on the segmentation of the target object from the background and achieve object recognition by describing and comparing all of the most significant geometric features of the three-dimensional object shape. Ho and Gibbins [10] utilized the shape index values and the standard deviation of neighboring points to measure ground changes, thereby identifying key points. A subsequent study [11] enhanced the expressive power of global features through multi-scale analysis.

Direct point cloud processing methods include the algorithm proposed by Tsai et al. [12], which converts RGB-D images into colored point clouds and uses the color signatures of the histogram of orientations (CSHOT) descriptor for object recognition. This approach circumvents several of the challenges associated with two-dimensional image processing, such as lighting and pose variations, by directly manipulating point cloud data. However, recognition methods based on colored point clouds and local texture shapes may fail in instances where color disparities are negligible or the object surface lacks texture. Furthermore, an enhanced point cloud feature for 3D object recognition based on normal vectors was proposed [13]. This method incorporates the distance information of neighboring points when estimating the normal vectors of RGB-D data point clouds, thereby enhancing recognition accuracy.

Deep learning methods have been widely applied to object recognition and six-dimensional (6D) pose estimation, particularly for handling complex scenes and improving recognition accuracy. Yu et al. [14] proposed a 3D object recognition method based on a view self-attention mechanism, which significantly improved classification accuracy and retrieval MAP by exploring local features of individual views and the connections between views. However, multi-view methods face practical limitations due to the field of view constraints of the acquisition equipment and are vulnerable to noise. Zhang et al. [15] proposed a lightweight deep learning algorithm that employs PointNet to extract global features for 3D visual recognition and optimizes classification and pose estimation through activation visualization. However, the generalization ability of this method in real-world scenarios still requires improvement. F. Yu [16] proposed a dual-channel convolutional neural network (CNN) method that integrates point cloud and multi-view image features, making it suitable for structured scenes and thereby effectively improving 3D object recognition accuracy.

In addressing the challenge of 6D pose estimation, Wen et al. [17] introduced the FoundationPose framework, which operates in both model-based and model-free settings. This framework utilizes neural implicit representations and is trained on large-scale synthetic data. As a result, FoundationPose demonstrated superior performance on multiple public datasets, highlighting its strong generalization capability. Nevertheless, the method’s reliance on the geometric shape of objects makes it susceptible to surface shape variations when handling non-rigid objects, such as dynamically changing skirts.

Wang et al. [18]. proposed the DenseFusion framework, designed for RGB-D data, which combines pixel-wise color and geometric features for 6D pose estimation. By integrating the PointNet architecture to extract depth information and incorporating an end-to-end iterative optimization module, DenseFusion significantly enhances robustness in occluded and cluttered scenes while also providing real-time inference capability. Building on DenseFusion, H. Li et al. [19] introduced DA2Net, which integrates a multi-channel attention mechanism to further improve the model’s adaptability to occlusion and lighting changes. DA2Net enhances pose estimation robustness by focusing on key features; however, the addition of more complex network structures increases computational complexity, which may affect real-time inference speed. Additionally, DenseFusion utilizes ResNet18 as an encoder to extract RGB color features, which may have limitations for objects with subtle textures and colors. Despite the numerous RGB or RGB-D data-based 6D pose estimation methods available, they still face challenges in processing real-world data, such as large rotations and translations, natural noise interference, and severe occlusion.

Point cloud data-based 6D pose estimation is a significant area of research. Jiang et al. [20] proposed a point cloud registration method based on the diffusion model in the Special Euclidean group in 3D (SE3), which greatly enhances pose estimation robustness and generalization ability through a denoising diffusion process, thereby offering a new approach to 6D pose estimation. However, this method typically demands substantial computational resources and training data, and its real-world application still requires further optimization. These research efforts indicate that although existing methods have achieved some success in theory and experiments, practical applications still face challenges that need to be addressed, including computational complexity, data dependence, and environmental factors.

Overall, the latest developments in the field of 3D object recognition are remarkable; however, the creation of 3D CAD models for non-rigid objects remains challenging, and the matching process is intricate. While deep learning methods demonstrate high accuracy, they require a substantial quantity of training samples, which presents a significant challenge, especially in industrial settings. The lack of transparency in the model’s internal mechanisms hinders the debugging process in the event of a fault. Robotic vision systems utilize image information to determine the spatial position of target objects and guide robots to perform specific actions. The process primarily involves handling input data, recognizing and localizing target objects [21], estimating object posture, detecting actuator grasping, and planning robotic grasping [22].

**Figure 2:**
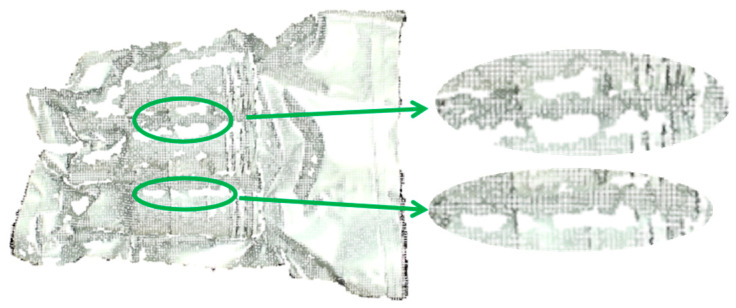
Holes in point cloud among target entities. The green circle denotes the regions of the target object where point cloud data are absent. The white section indicates the missing point cloud data, while the silver region signifies the point cloud of the packaging.

The primary objective of this paper is to employ robotic vision guidance technology to grasp the NMSDP, as illustrated in Figure 1b. The focus of this study is the NMSDP product, which is packaged in an aluminum foil bag and vacuum-sealed. The product exhibits the following characteristics: the packaging surface is non-rigid and irregular, with a significant front skirt portion that appears flat or variously raised. The middle section contains three target bodies with slight flotation, which highlights the overall outline of the product’s main body. The tail skirt section is highly complex, featuring raised edges, curled edges, depressions, and various irregular shapes, which complicates segmentation. As demonstrated in Figure 2, cavities in the point cloud data are inevitable during the packaging point cloud imaging process due to the influence of the aluminum foil material and shape. These cavities are generally distributed along the concave edges near the target bodies, and the surface quality is uneven, which affects the accuracy of pose estimation. Therefore, this paper conducts a study on 6D pose estimation for this type of packaging to enable precise robotic grasping.

In summary, the primary contributions of this paper are as follows:

1. A pre-segmentation method based on the minimum bounding box was employed to filter out the wrinkled and lifted packaging blades and skirts, reducing the impact of packaging instability on target object segmentation.
2. A directional filter was combined with Euclidean clustering and Principal Component Analysis (PCA) principal axis feature selection to segment the target body. Through iterative operations, the accuracy of target body segmentation and extraction was improved.
3. A multi-target body feature fusion pose estimation method was proposed to mitigate the impact of point cloud cavities, enabling accurate estimation of the target’s 6D pose.

The structure of this paper is as follows:

The second section introduces the configuration of the robotic grasping system. The third section presents the pose estimation method, including pre-segmentation based on the minimum bounding box, improved minimum bounding box normal vector direction establishment for filtering direction determination, and target extraction using directional filters combined with Euclidean clustering and PCA principal features, as well as multi-feature fusion pose estimation. The fourth section discusses the selection of experimental parameters and analyzes the results, and the fifth section concludes the findings.

## 2. Vision System Design

**Figure 3:**
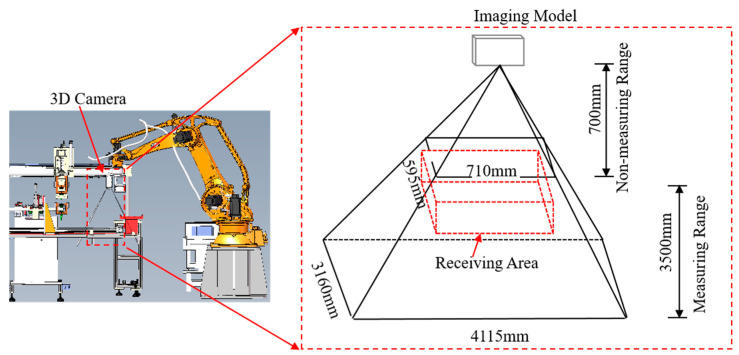
Product cutting system and 3D camera system. The left image shows the front view of the CAD drawing, representing the layout of the on-site mechanism distribution. The right image is a schematic of the 3D camera system intended to illustrate the working range of the 3D camera.

As illustrated in Figure 3, the system is built upon a palletizing robot, forming the vision-guided NMSDP packaging system. The system consists of three main components: a palletizing robot, a product transportation platform, and a 3D camera. The palletizing robot employed is an ESTUN four-axis robot ER60-2000, featuring a working radius of 2 m and a load capacity of 60 kg. At the robot’s end effector, a sponge suction cup measuring 294 mm × 130 mm × 70 mm is mounted via a fixture. Opposite the suction cup, an RGB-D camera model FM811-IX-E1 is installed. The RGB-D camera, comprising an infrared laser emitter, an infrared camera, and an RGB camera, projects a predefined infrared pattern into the object’s surface, and the infrared camera captures the deformed pattern reflected back due to the object’s shape. By analyzing the pattern deformation, the camera computes depth data. The depth image resolution is 1280 × 960 pixels, with a depth accuracy of 1.17 mm at an 800 mm working distance. The camera’s effective depth measurement range spans from 700 mm to 3500 mm, covering a far field of view of 4115 mm × 3160 mm.

In the factory environment studied, traditional cameras are often affected by reflections and shadows, which compromise object recognition and localization accuracy. In contrast, the RGB-D cameras mitigate these issues by providing reliable depth information and accurate spatial positioning, significantly enhancing localization robustness. After production, the NMSDP is vacuum-sealed in an aluminum foil bag, each with dimensions of 160 mm × 280 mm × 50 mm; the packaged products are subsequently transported via a conveyance pallet for further processing.

## 3. Methods

### 3.1. Overview

**Figure 4:**
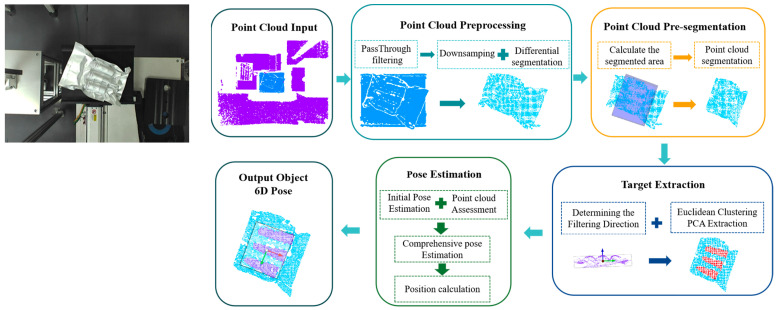
The overall process framework for 6D pose estimation of the NMSD Pack. The image on the left shows the RGB image acquired by the camera during an actual operational scenario.

As illustrated in Figure 4, the overall framework developed for the 6D pose estimation of the NMSDP consists of three distinct procedures: pre-segmentation, target extraction, and pose estimation. First, pre-segmentation is performed based on the minimum bounding box. Second, target extraction is achieved by combining directional filtering with Euclidean clustering and PCA-based principal features. Third, feature fusion is applied for pose estimation of multiple target bodies.

Before extracting the target body and estimating its pose, the input point cloud data undergo several preprocessing steps, including pass-through filtering, point cloud downsampling, and point cloud difference-based segmentation. These steps aim to effectively isolate the NMSDP product from the environmental background.

1. Preliminary segmentation: A modified minimum bounding box is employed as the segmentation area to eliminate wrinkled and lifted packaging blades and skirts. This method minimizes the impact of the packaging instability on target body segmentation.
2. Target extraction: A directional filter is utilized in combination with Euclidean clustering and PCA-based principal features to identify and segment the target body. Through iterative processing, the segmentation accuracy is progressively enhanced.
3. Pose estimation: For each extracted target body, PCA principal feature vectors and minimum bounding box vectors are computed. The positions of the target bodies, the relationships between vectors, and standard deviation of point cloud curvature are analyzed to assess the quality of the point cloud data. A dynamic weighting coefficient approach is applied to integrate these features, enabling a comprehensive estimation of the packaged target’s pose.

### 3.2. Region Segmentation Based on the Minimum Bounding Box Variant

**Figure 5:**
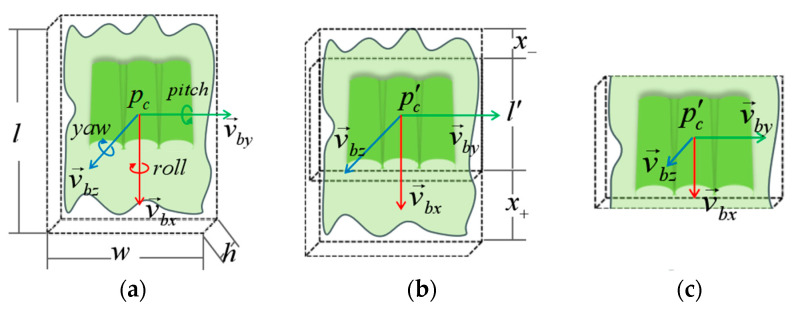
Minimum bounding box Segmentation. (**a**) Minimum bounding box encloses the object; (**b**) modify the size of the minimum bounding box; (**c**) segment the object.

As shown in Figure 5a, the minimum bounding box is defined for a set of points. The objective of the minimum bounding box algorithm is to identify a rectangular prism with the smallest volume that can fully encompass all points, while the orientation of the bounding box is determined based on the geometric shape of the target.

In the process of pre-segmenting the packaging point cloud, the overall volume size and direction of the packaging point cloud are determined based on the minimum bounding box. The bounding box model is represented as follows:

(1) $$\begin{array}{c} B_{c}\left(x_{c},y_{c},z_{c},roll,pitch,yaw,l,w,h\right) \end{array}$$

The center coordinates$\;\left(x_{c},y_{c},z_{c}\right)\;$of the bounding box are defined as $p_{c}$. The spatial attitudes of the bounding box, represented by Euler angles, are denoted by roll,pitch, and yaw. The length, width, and height of the bounding box are represented by l,w,h, respectively. The pose of the bounding box can be transformed to obtain its coordinate system, with the coordinate axis represented by three 3D vectors ${\vec{v}}_{bx}\left(a_{x},b_{x},c_{x}\right)$, ${\vec{v}}_{by}\left(a_{y},b_{y},c_{y}\right)$, ${\vec{v}}_{bz}\left(a_{z},b_{z},c_{z}\right)$. As shown in Figure 5b, to mitigate the impact of the front blade and the rear skirt on subsequent target segmentation, regional segmentation is applied to segment these two parts. The position and size of the region are calculated and adjusted based on the current position and size of the bounding box. The adjustment rules are represented by the following formulas:

(2) $$\begin{array}{c} d=x_{+}-x_{-} \end{array}$$

(3) $$\begin{array}{c} l^{\prime }=l-x_{+}-x_{-} \end{array}$$

In the given context, the adjusted length is represented by d, while $x_{+}$ and $x_{-}$ denote the positive and negative changes, respectively, along the direction of ${\vec{v}}_{bx}$. The adjusted rectangular prism length is denoted by $l^{\prime }$. The components of the new center coordinates of rectangular cuboid $p_{c}^{\prime }\left(x_{c}^{\prime },y_{c}^{\prime },z_{c}^{\prime }\right)\;$ are expressed as follows:

(4) $$\begin{array}{c} \left\{\begin{array}{c} x_{c}^{\prime }=x_{c}-\frac{d}{2}\cdot a_{x}\\ y_{c}^{\prime }=y_{c}-\frac{d}{2}\cdot b_{x}\\ z_{c}^{\prime }=z_{c}-\frac{d}{2}\cdot c_{x} \end{array}\right. \end{array}$$

Following the described methodology, the newly formulated rectangular prism is defined as

(5) $$\begin{array}{c} B_{c}^{\prime }\left(x_{c}^{\prime },y_{c}^{\prime },z_{c}^{\prime },roll,pitch,yaw,l^{\prime },w,h\right) \end{array}$$

**Figure 6:**
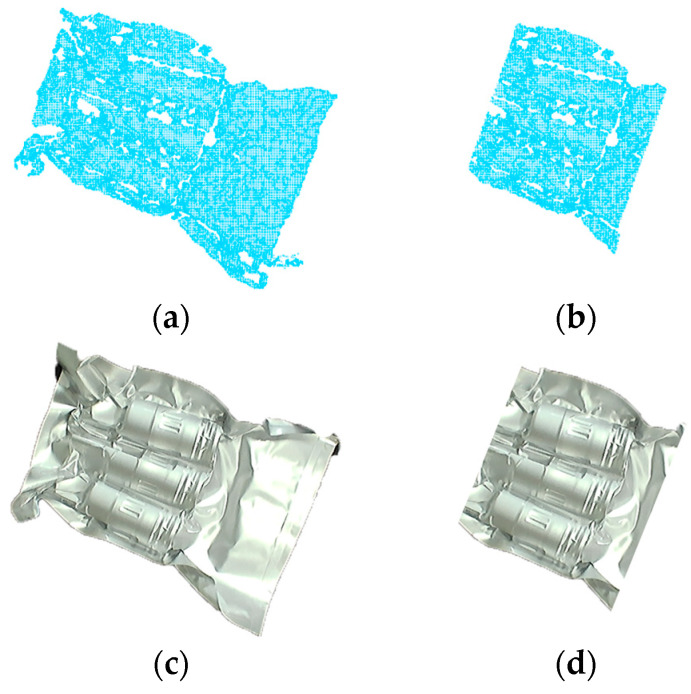
Point cloud pre-segmentation. (**a**) Point cloud ∁ before pre-segmentation; (**b**) point cloud $C^{\prime }$ after pre-segmentation; (**c**) corresponding RGB image of the point cloud in (**a**); (**d**) corresponding RGB image of the point cloud in (**b**).

Using this new rectangular prism as the segmentation area, the front and rear skirts are removed, leaving only points within $B_{c}^{\prime }\;$(Figure 5c). As shown in Figure 6b, this process produces a point cloud $C^{\prime }$, which contains only the central section of the target, including all the point clouds of the three target bodies.

### 3.3. Target Extraction Based on Directional Filtering Combined with Euclidean Clustering and PCA Principal Features

Despite the efficacy of the regional segmentation method in removing significant noise from the front and rear skirts, it is still unable to effectively segment the target body from the packaging. This paper proposes an iterative segmentation method that combines directional filtering with Euclidean clustering, enabling effective separation of the target body from the packaging background. The method employs a directional filter for the preliminary segmentation of the packaging bag, followed by clustering combined with PCA feature vectors to extract the target.

A directional filter operates along a specific direction, removing noise or segmenting point clouds within that direction. Selecting the appropriate filtering direction is crucial, as any deviation may result in either under-segmentation or over-segmentation of the target point cloud, thereby affecting segmentation accuracy. Since the surface of the target body is not parallel to the camera plane, the *z*-axis is unsuitable as the filtering direction. Moreover, the rotated minimum bounding box does not closely align with the object’s surface, making its normal vector also unsuitable as the filtering direction.

**Figure 7:**
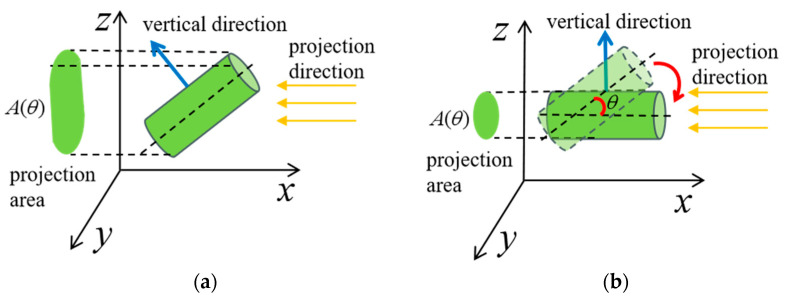
Target rotation projection. (**a**) Target general projection area pose; (**b**) target minimum projection area pose.

The present paper proposes a refined minimum bounding box algorithm, aiming to enhance its compatibility with the surface of the target object and determine the optimal filtering direction. The underlying principle of the algorithm is described as follows:

As illustrated in Figure 7, let $A\left(\theta \right)$ denote the projected area of an object in a specific projection direction. It has been demonstrated that there exists a rotation angle θ such that $A\left(\theta \right)$ is minimal when the object is rotated, such that the object’s surface normal vector becomes parallel to the coordinate system’s *Z*-axis, and the XY plane of the coordinate system is parallel to the object’s surface. For a continuously rotating object, the projection of the object onto the side and front planes of the rectangular solid $B_{c}^{\prime }$, will result in a minimum projected area. The rotation from the initial projection direction to this minimum area is denoted as $\theta_{min}$. Alternatively, instead of rotating the object, the rectangular solid can be rotated within its own coordinate system. Here, ${\vec{\;v}}_{by}\;$ and $\;{\vec{v}}_{bx}\;$ are the normal vectors of the side and front planes, respectively. The following steps are performed by rotating the box $B_{c}^{\prime }$ first in Roll and then in Pitch:

1. The point cloud is projected along the direction of ${\vec{v}}_{by}$ onto the side plane of the rectangular solid.
2. Deduplication processing is performed on the projected point cloud.
3. The number of points in the projected point cloud is calculated.
4. The parameter $\;\theta_{r}$ is updated by rotating $\theta_{r}$ degrees around $\;{\vec{v}}_{bx}$, and steps 1 to 3 are repeated to minimize the number of projected points.

**Figure 8:**
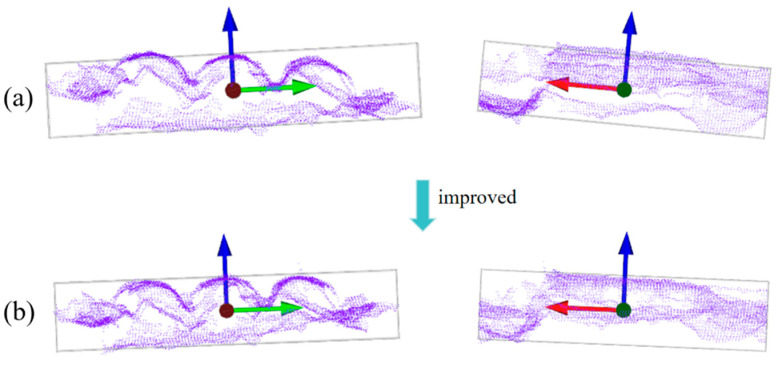
Improved minimum bounding box. (**a**) Front and side of the minimum bounding box: The blue arrow in the figure represents the Z-axis direction of the minimum bounding box, the green arrow represents the Y-axis direction, and the red arrow represents the X-axis direction; (**b**) front and side of the improved minimum bounding box.

In a similar manner, the point should be projected along the direction of ${\vec{v}}_{bx}$ onto the front plane, and $\theta_{p}$, is rotated around ${\vec{v}}_{by}$. The aforementioned steps are repeated to obtain the optimal $\theta_{p_{min}}$ value. As shown in Figure 8b, when the rectangular solid coordinate system is rotated by $\theta_{r_{min}}$ and $\theta_{p_{min}}$ in the Roll and Pitch order, the rectangular solid better fits the surface of the packaged object. In this state, ${\vec{v}}_{bz}$ is considered to be the direction perpendicular to the surfaces of the three target bodies, thus serving as the filtering direction for the directional filter.

In pursuit of the optimal $\theta_{r_{min}}\;$ and $\;\theta_{p_{min}}$, this paper introduced a heuristic search algorithm that combined the strengths of gradient descent and binary search methods. This approach facilitates the efficient identification of the optimal solution while reducing the likelihood of getting trapped in local optima. Specifically, let *K* ∈ {0, 1, 2, ⋯, *N*} denote the iteration index:

(6) $$\begin{array}{c} \theta_{L,K}=\theta_{K}-\Delta \theta_{K},\;\;\;\;\;\;\theta_{L,K}\in \left[-\pi ,\;\;\;\;\;\pi \right] \end{array}$$

(7) $$\begin{array}{c} \theta_{R,K}=\theta_{K}+\Delta \theta_{K},\;\;\;\;\;\;\theta_{R,K}\in \left[-\pi ,\;\;\;\;\;\pi \right] \end{array}$$

(8) $$\begin{array}{c} \theta_{new,K}=\mathrm{argmin}(A\left(\theta_{K}\right),A(\theta_{L,K}),A(\theta_{R,K})) \end{array}$$

(9) $$\begin{array}{c} \left\{\begin{array}{c} \Delta \theta_{K+1}=\Delta \theta_{K}/2,\;\theta_{\mathrm{new},K}=\theta_{K}\\ \Delta \theta_{K+1}=\Delta \theta_{K},\;\theta_{\mathrm{new},K}\neq \theta_{K} \end{array}\right. \end{array}$$

(10) $$\begin{array}{c} \left\{\begin{array}{c} \theta^{*}=\theta_{new,K}\;\;\Delta \theta_{K}<\varepsilon \\ \theta_{K+1}=\theta_{new,K}\;\Delta \theta_{K}>\varepsilon \end{array}\right. \end{array}$$

At each *K*-th iteration, $\theta_{K}$ represented the current rotation angle while $\theta_{L,K\;}\;$ and $\theta_{R,K}$ represented the left and right rotation angles, respectively. The search step size at the *K*-th iteration was denoted by $\Delta \theta_{K}$, and $\theta_{new,K}$ corresponded to the angle that yielded the minimal projected area at that iteration. The optimal rotation angle sought upon convergence was denoted as ${\;\theta }^{*}$. The optimal values of $\theta_{r_{min}}$ and $\theta_{p_{min}}$ were determined by initializing the process with the initial angle $\theta_{0\;}$ and the initial search step size $\Delta \theta_{0}$ at the first iteration.

Once the optimal filtering direction is determined by setting the threshold for the directional filter, the packaging point cloud is segmented along the direction of ${\vec{v}}_{bz}$. This enables the separation of the target body from the packaging background. However, a fixed threshold is inadequate for sufficiently separating the three target bodies from the packaging background in all input packaging objects. Consequently, identifying an adaptive threshold becomes imperative. The paper utilizes Euclidean clustering and PCA feature vector selection to verify whether the target body is sufficiently separated from the packaging background, to assess if the current segmentation threshold is optimal. Euclidean clustering classifies the segmented point cloud into distinct blocks based on the point distance, filtering out those that do not meet the required point count. PCA calculates the feature vectors for each block and extracts the target object by analyzing the similarity of these feature vectors. The following formulas outline the process of using Euclidean clustering and PCA feature vectors to filter the target:

(11) $$\begin{array}{c} C=\left\{C_{k}\left|\left|C_{k}\right|\geq n_{min}\;\;\;\;\;k=1,2,\ldots ,K\right.\right\} \end{array}$$

In the equation, $n_{min}$ represents the minimum threshold for the number of points, $C_{k}$ is the *K*-th point cloud block in the set of all point cloud clusters, and C is the set of all point cloud clusters meeting the point count requirement. Let ${\vec{v}}_{m}$, ${\vec{v}}_{n}$, and ${\vec{v}}_{o}$ represent the principal feature vectors of the point cloud clusters $C_{m}$, $C_{n}$, and $C_{o}$, respectively. The cosine similarity between each pair of feature vectors is calculated as follows:

(12) $$\begin{array}{c} \left\{\begin{array}{c} \cos\left(\alpha_{mn}\right)=\frac{{\vec{v}}_{m}\cdot {\vec{v}}_{n}}{\left\|{\vec{v}}_{m}\right\|\;\left\|{\vec{v}}_{n}\right\|}\\ \cos\left(\alpha_{mo}\right)=\frac{{\vec{v}}_{m}\cdot {\vec{v}}_{o}}{\left\|{\vec{v}}_{m}\right\|\;\left\|{\vec{v}}_{o}\right\|}\\ \cos\left(\alpha_{no}\right)=\frac{{\vec{v}}_{n}\cdot {\vec{v}}_{o}}{\left\|{\vec{v}}_{n}\right\|\;\left\|{\vec{v}}_{o}\right\|} \end{array}\right. \end{array}$$

Here, $\alpha_{mn},\alpha_{mo},and\;\alpha_{no}$ are the angular separations between the feature vectors of the corresponding point cloud clusters. The similarity threshold is defined by $\cos(\alpha_{min})$, which is the minimum acceptable cosine similarity value. The set P contains all valid triplets of point cloud clusters $\left(C_{m},C_{n},C_{o}\right)$ that satisfy the following conditions:

(13) $$\begin{array}{c} P=\left\{\left(C_{m},C_{n},C_{o}\right)\;\in C\times C\times C\left|\begin{array}{c} n\neq m,o\neq m,o\neq n,\\ \cos(\alpha_{mn})\geq \cos(\alpha_{min}),\\ \begin{array}{c} \cos(\alpha_{mo})\geq \cos(\alpha_{min}),\\ \cos(\alpha_{no})\geq \cos(\alpha_{min}) \end{array} \end{array}\right.\right\} \end{array}$$

In this formula, C×C×C represents the cartesian product of all possible triplets of point cloud clusters, meaning three-point cloud clusters, $\;C_{m}$, $C_{n}$, and $C_{o},$ are selected from the set C to form a triplet.

**Figure 9:**
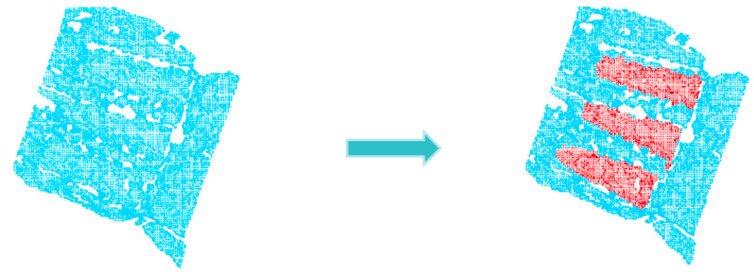
Extract three target entities. The left side of the figure shows the point cloud to be extracted after pre-segmentation, while the right side displays the point cloud after extraction. The red regions indicate the successfully extracted point clouds of the three target objects.

The screening method applied to extract the target body determined the current threshold to be optimal, as indicated by the number of target bodies extracted. As shown in Figure 9, if the threshold is further adjusted and iteratively applied for segmentation and screening, the optimal threshold can be identified, thereby enabling effective separation of the target body from the packaging background.

### 3.4. Pose Estimation for Multiple Target Bodies Based on Feature Fusion

**Figure 10:**
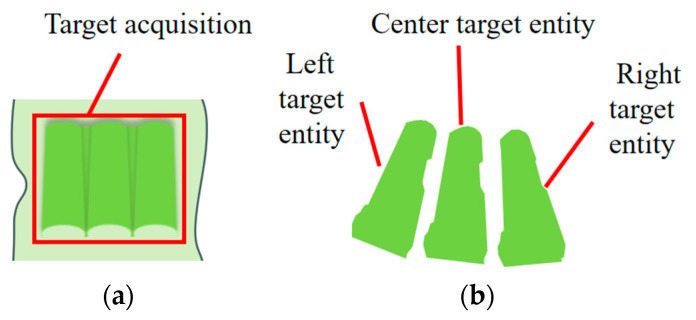
Target and target entity in packaging. (**a**) Target in packaging; (**b**) extracted target entity.

As shown in Figure 10a, the target consists of three molecular sieve packages, which constitute the main structural component of the packaging. Figure 10b depicts the extracted target bodies from the packaging in the point cloud processing stage, categorized into left, middle, and right target bodies. These three target bodies collectively represent the primary focus of this study. In the pose estimation stage, this paper estimates the poses of individual target bodies, which collectively determine the pose of the overall target, rather than directly estimating the pose of the entire packaging.

However, the extracted target body may have incomplete local edge point clouds, leading to irregularities in local edges. This can adversely affect the target’s pose estimation. To address this, this paper proposes a weighted pose estimation method for multiple target bodies, aiming to minimize errors caused by uncertainties. The weighting process incorporates features such as the standard deviation of edge curvature, the discrepancy between the minimum bounding box principal vectors and the PCA principal feature vectors of the target bodies to evaluate the confidence of the pose estimation, and the positional relationship between the target bodies.

The prominent overall shape and contour features of the target body make the minimum bounding box a viable method for calculating its position and orientation in space. However, the minimum bounding box method is sensitive to directional fluctuations and deviations when edge points are missing. Given the shape of the target body, PCA features, which describe the overall structure, can partially compensate for the directional limitations inherent to the minimum bounding box.

Missing edge point clouds in target objects are a common occurrence. Therefore, estimating the pose of the middle target body alone as the overall target pose may lack accuracy. Nonetheless, the pose of the middle target body remains crucial. To mitigate errors caused by missing point clouds, the poses of the left and right target bodies are introduced as auxiliary information. Ideally, the left and right target bodies would maintain a specific angle relative to the middle target body and exhibit symmetric distribution. In practice, however, these bodies have some degree of freedom, requiring adjustments to accommodate varying angular positions.

**Figure 11:**
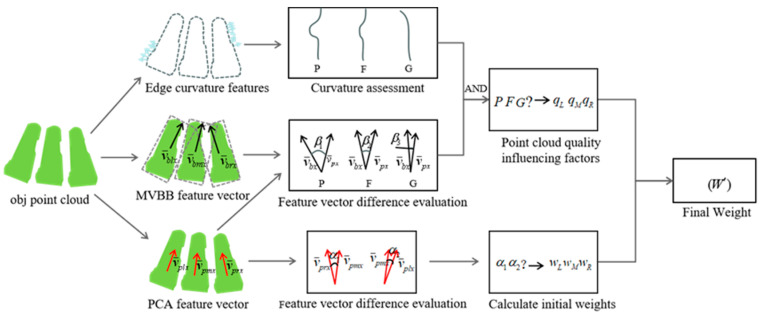
Feature fusion determination process.

As shown in Figure 11, this paper employs a dynamic weighting coefficient method to evaluate the poses of the target bodies at various positions. The determination of the weighting coefficients is as follows: Firstly, a principle based on position importance assessment is established. Specifically, in typical scenarios, the weighting coefficient for the pose of the middle target body is assigned slightly higher values compared to those of the side targets. Secondly, the paper adopts a segmented weighting approach, where the difference in the PCA principal feature directions between the middle target body and the side target bodies are used to determine the weighting coefficients for each target body.


(14) $$\begin{array}{c} W\left(w_{M},w_{L},w_{R}\right)=\left\{\begin{array}{c} \left(w_{M1},w_{L1},w_{R1}\right),\;\alpha_{1}<\alpha ,\alpha_{2}<\alpha \\ \left(w_{M2},w_{L2},w_{R2}\right),\alpha_{1}<\alpha <\alpha_{2}\\ \left(w_{M3},w_{L3},w_{R3}\right),\alpha_{2}<\alpha <\alpha_{1}\\ \left(w_{M4},w_{L4},w_{R4}\right),\alpha_{1}>\alpha ,\alpha_{2}>\alpha \end{array}\right. \end{array}$$

(15) $$\begin{array}{c} \forall i\in \left\{1,2,3,4\right\},\;w_{Mi}+w_{Li}+w_{Ri}=1 \end{array}$$

In the given equation, $\alpha_{1}$ and $\alpha_{2}$ represent the feature angles between the middle target body and the left and right-side target bodies, respectively. α is the feature angle threshold, and $w_{M}$, $w_{L}$, and $w_{R}$ are the initial weight coefficients for the middle target body *M* and the left and right-side target bodies *L* and *R*, respectively. Note that the target bodies obtain weight combinations based on their positional feature angle relationships, allowing for the calculation of the initial pose.

In order to enhance the stability of pose estimation, a reliability confidence metric is introduced to assess the target body’s pose, with two criteria for scoring the pose confidence. The first criterion compares the principal axis direction of the bounding box with the direction obtained from PCA. The difference between these directions reveals the partial status of the target object. Three cases are considered: When the directional difference is large, the target object shows relatively slender local protrusions, indicating low confidence (*P*). When the difference is moderate, the target shows a conical shape due to partial point cloud missing, resulting in a fair confidence score (*F*). When the difference is small, the target is relatively complete, indicating high confidence (*G*).

(16) $$\begin{array}{c} q_{deg}=\left\{\begin{array}{c} P,\;\beta_{max}<\beta \;\\ F,\;\beta_{min}<\beta <\beta_{max}\\ G,\;{\beta <\beta }_{min} \end{array}\right. \end{array}$$

In the formula, β  represents the angular difference between the two principal axis directions, while $\beta_{min}$ and $\beta_{max}$ denote the threshold for small and large angular differences, respectively. Note that this method may not fully account for targets with local point cloud voids. To improve the evaluation, an additional scoring criterion is introduced: the concavity and convexity of the point cloud edge. The concavity and convexity are characterized by attributes such as their degree, range, and locations. The quality of the point cloud is measured by the standard deviation of the curvature at the edge; lower standard deviations indicate higher confidence in the pose.

(17) $$\begin{array}{c} q_{k}=\left\{\begin{array}{c} P,\;\gamma_{max}<\gamma \;\\ F,\;\gamma_{min}<\gamma <\gamma_{max}\\ G,\;{\gamma <\gamma }_{min} \end{array}\right. \end{array}$$

In the equation, γ denotes the standard deviation of the curvature along the point cloud edge while $\gamma_{min}$ and $\gamma_{max}$ represent the low and high standard curvature thresholds, respectively. The point cloud quality is evaluated through an integrated scoring system based on these two criteria. The comprehensive evaluation score for the target bodies *M*, *L*, and *R* is given by

(18) $$\begin{array}{c} \left\{\begin{array}{c} q_{M}=q_{Mdeg}+q_{Mk}\\ q_{L}=q_{Ldeg}+q_{Lk}\\ q_{R}=q_{Rdeg}+q_{Rk} \end{array}\right. \end{array}$$

The total quality influence factor, Q, is calculated as

(19) $$\begin{array}{c} Q=w_{L}\cdot q_{L}+w_{M}\cdot q_{M}+w_{R}\cdot q_{R} \end{array}$$

The final dynamic weighting coefficients are

(20) $$\begin{array}{c} \left\{\begin{array}{c} w_{L}^{\prime }=\frac{w_{L}\cdot q_{L}}{Q}\\ w_{M}^{\prime }=\frac{w_{M}\cdot q_{M}}{Q}\\ w_{R}^{\prime }=\frac{w_{R}\cdot q_{R}}{Q} \end{array}\right. \end{array}$$

Here, $w_{M}^{\prime }$, $w_{L}^{\prime }$, and $w_{R}^{\prime }$ represent the final dynamic weights for the poses of the target bodies *M*, *L*, and *R*, respectively. These weights are used to integrate pose estimations into the final pose. The roll, pitch, and yaw angles are calculated as

(21) $$\begin{array}{c} \left\{\begin{array}{c} roll=w_{L}^{\prime }\cdot r_{L}+w_{M}^{\prime }\cdot r_{M}+w_{R}^{\prime }\cdot r_{R}\\ pitch=w_{L}^{\prime }\cdot p_{L}+w_{M}^{\prime }\cdot p_{M}+w_{R}^{\prime }\cdot p_{R}\\ yaw=w_{L}^{\prime }\cdot y_{L}+w_{M}^{\prime }\cdot y_{M}+w_{R}^{\prime }\cdot y_{R} \end{array}\right. \end{array}$$

These angles define the target’s spatial orientation. To facilitate a comparison of experimental results, the Euler angles are converted into quaternions as follows:

(22) $$\begin{array}{c} \left\{\begin{array}{c} q_{roll}=\cos\left(\frac{roll}{2}\right)+\sin\left(\frac{roll}{2}\right)i_{x}\\ q_{pitch}=\cos\left(\frac{pitch}{2}\right)+\sin\left(\frac{pitch}{2}\right)i_{y}\\ q_{yaw}=\cos\left(\frac{yaw}{2}\right)+\sin\left(\frac{yaw}{2}\right)i_{z} \end{array}\right. \end{array}$$

The final quaternion q is given by

(23) $$\begin{array}{c} q=q_{yaw}\cdot q_{pitch}\cdot q_{roll} \end{array}$$

In this context, $q_{yaw}$, $q_{pitch}$, and $q_{roll}$ are the quaternions representing the yaw, pitch, and roll rotations, respectively. The imaginary coefficients $i_{x}$, $i_{y}$, and $i_{z}$ are used to represent the rotation axes. The final quaternion q represents the Euler orientation of the target.

**Figure 12:**
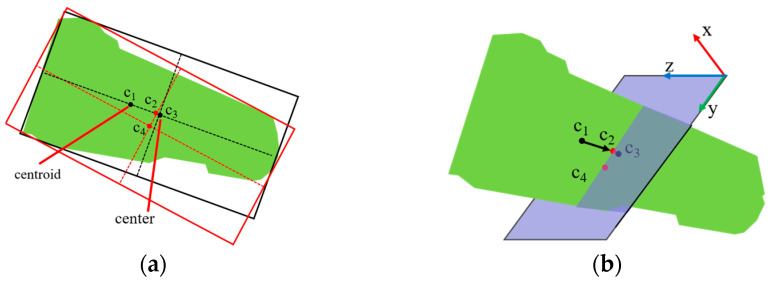
Center position compensation model. (**a**) Minimum bounding box enclosing object, the red frame represents the minimum bounding box surrounding the object, the black frame represents the ideal minimum bounding box, c1 is the centroid of the object, c2 is the intersection point of the short axis of the red frame and the long axis of the black frame, c3 is the ideal center of the object, and c4 is the center of the bounding box; (**b**) the long axis of the attitude intersects the plane of the bounding box.

The positional center of the object is determined as follows. As shown in Figure 12a, the red frame represents the minimum bounding box surrounding the object, the black frame represents the ideal minimum bounding box, *c*_1_ is the centroid of the object, *c*_2_ is the intersection point of the short axis of the red frame and the long axis of the black frame, *c*_3_ is the ideal center of the object, and *c*_4_ is the center of the bounding box. During position estimation, the minimum bounding box method generally calculates the object center. However, due to the object’s shape, there is a deviation between the direction of the minimum bounding box and the actual axis, which causes the center to deviate from the true object center. Therefore, this paper introduces the axis direction of the final pose to adjust the bounding box orientation and adjusts it based on the effective length of the object inside the packaging. This method stabilizes the bounding box center, even when the target point cloud length varies.

As shown in Figure 12b, to refine the position estimation, a bounding box center plane is constructed. The plane’s normal vector is aligned with the *x*-axis of the bounding box, and the center point *c*_4_ serves as a point on the plane. The plane is constructed using the point-normal form. Then, the centroid *c*_1_ of the initial target body point cloud is used as the starting point, and the vector along the long axis direction of the final pose intersects with the bounding box center plane. The intersection point *c*_2_ is considered the approximate center of the target point cloud. This method provides a more accurate estimation of the packaged object’s overall pose.

## 4. Experiments and Analysis

### 4.1. Experimental Preparation

The experimental system consists of a 3D camera, an industrial PC, an industrial robot (equipped with a sponge suction cup at the end), a programmable logic controller (PLC), and a conveyor device. Visual Studio 2022 is used as the image processing development platform, and the algorithms are written in C++. RobotVisionSuite version 1.5.1129 is used as the software execution platform, configured with an i7-12700H CPU model and RTX3060 GPU running on a Windows 11 system.

The initial experimental data consist of 48 sets of point cloud data for robotic grasp, collected by the on-site mounted 3D camera. Prior to this experiment, hand–eye calibration between the robot and the 3D camera was performed.

**Figure 13:**
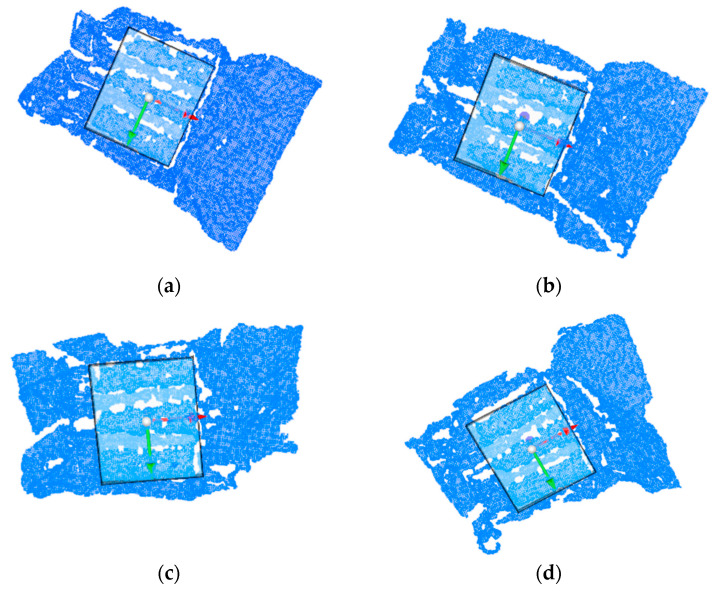
Ground truth box. (**a**–**d**) Truth box annotation for partial data.

As shown in Figure 13, to rigorously assess the accuracy of the target posture estimation results, manual posture annotation was performed on all point cloud datasets. This involved creating a standard parallelepiped, where the dimensions of the upper surface of the packaging target were physically measured to determine the cuboid’s length, width, and height as 130 mm × 110 mm × 10 mm. By incrementally adjusting the cuboid’s position and orientation, we ensured that the primary point cloud of the target was fully encapsulated and perfectly aligned. The central position and orientation of this cube were taken as the true position of the target point cloud. The accuracy of the algorithm was verified by comparing the target postures calculated by the algorithm with those manually annotated as the true postures.

**Figure 14:**
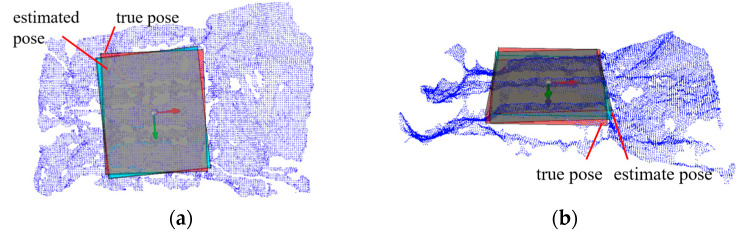
Estimated pose vs. true pose comparison. (**a**) Differences in yaw angle between estimated and true pose. The red frame represents the manually annotated frame, and the blue frame is generated based on the target pose estimated by the method described in this paper; (**b**) differences in roll and pitch angles between estimated and true pose.

Figure 14 shows a comparison between the pose estimation method proposed in this paper and the manually annotated true pose. The red frame represents the manually annotated frame, and the blue frame is a cube generated with dimensions matching the manually annotated frame based on the target pose estimated by the method described in this paper.

In the experiment, the product body is not in the center of the bag. When performing preliminary segmentation on the packaging skirt, selecting the appropriate segmentation length is crucial. A segmentation length that is too short will have a limited effect, while one that is too long will miss parts of the target, affecting subsequent segmentation and pose estimation. To achieve the expected segmentation effect without affecting the target body, this paper selects segmentation length parameters $x_{+}$ = 0.08 m and $x_{-}$ = 0.04 m through experiments on all data. This approach ensures effective segmentation while providing a margin to avoid interfering with subsequent target segmentation.

### 4.2. Discussion on Parameters for Point Cloud Extraction of Target Body

When extracting the point cloud of the target body, three key parameters must be considered: the filtering direction, the clustering point count threshold, and the main feature similarity threshold.

#### 4.2.1. Discussion on the Initial Search Angle Step

The accuracy of the filtering direction, as the basis for the segmentation of the target point cloud, determines whether the target can be fully segmented. The filtering direction is defined by the normal vector ${\vec{v}}_{bz}$ of the bounding box surface. From the improved bounding box algorithm, it is evident that the parameters affecting the bounding box are the initial angle $\theta_{0}$ and the initial search step size $\Delta \theta_{0}$, with $\theta_{0}$ representing the current pose of the bounding box before improvement. Therefore, the primary focus is on the effect of the initial search step size $\Delta \theta_{0}$ on the bounding box. If the angular step size is too large, the search efficiency decreases, which impacts computational speed; if the angular step size is too small, the search results may deteriorate, potentially falling into local optimal solutions.

**Figure 15:**
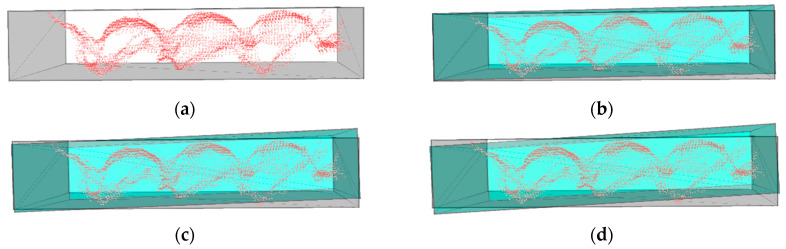
Effect with different initial search angle step sizes. (**a**) Initial bounding box; (**b**) $\Delta \theta_{0}$ = 0.5°; (**c**) $\Delta \theta_{0}$ = 1, 5, 7°; (**d**) $\Delta \theta_{0}$ = 3, 9°.

To balance computational accuracy and algorithmic efficiency, this paper sets the initial search angle step sizes to 0.5°, 1°, 3°, 5°, 7°, and 9° and conducts experiments on 48 sets of point cloud data. The parallelism between the bounding box surface and the target point cloud surface under different parameter settings is calculated. Table 1 presents the results, showing the greatest parallelism between the bounding box and the target point cloud for various parameters. Based on the number of results with higher parallelism, the optimal search step size $\Delta \theta_{0}$ = 3° is determined, Figure 15 shows the effects under different $\Delta \theta_{0}$ parameters.

#### 4.2.2. Discussion on Thresholds for Clustering and Feature Similarity

During the process of directional filtering and iterative segmentation for extracting the main body point cloud of a target, selecting appropriate thresholds for clustering point count and main feature similarity was crucial for accuracy target extraction. In the experiment, a clustering point threshold of $n_{min}$ = 400 was chosen to effectively filter out small to medium-sized point cloud blocks while preserving the target. Furthermore, a main feature similarity angle threshold of $\alpha_{min}=15{}^{\circ}$ was selected to ensure that all target point clouds met the similarity requirements while filtering out point cloud blocks with excessive angular deviations. Table 2 illustrates the effectiveness and reliability of this method in extracting the main bulk of the target point cloud.

### 4.3. Experimental Validation of Pose Estimation

#### 4.3.1. Discussion on Initial Weight Parameter Selection

In the target pose estimation process, the angular difference α of the three main axes of the target’s main body was used to determine the initial weights. An angular threshold that was too large failed to reflect differences between targets, while a threshold that was too small exaggerates these differences. Thus, selecting an appropriate angular threshold parameter was crucial for determining the initial weights. Experiments were conducted with angular thresholds of α = 1.5°, α = 2°, and α = 3°, corresponding to weight distributions of 0.3, 0.2, and 0.5, respectively. The computed poses were compared to true poses to verify the algorithm’s accuracy. To ensure consistency in the comparison, the three Euler angles of the target were converted to quaternion form.

**Figure 16:**
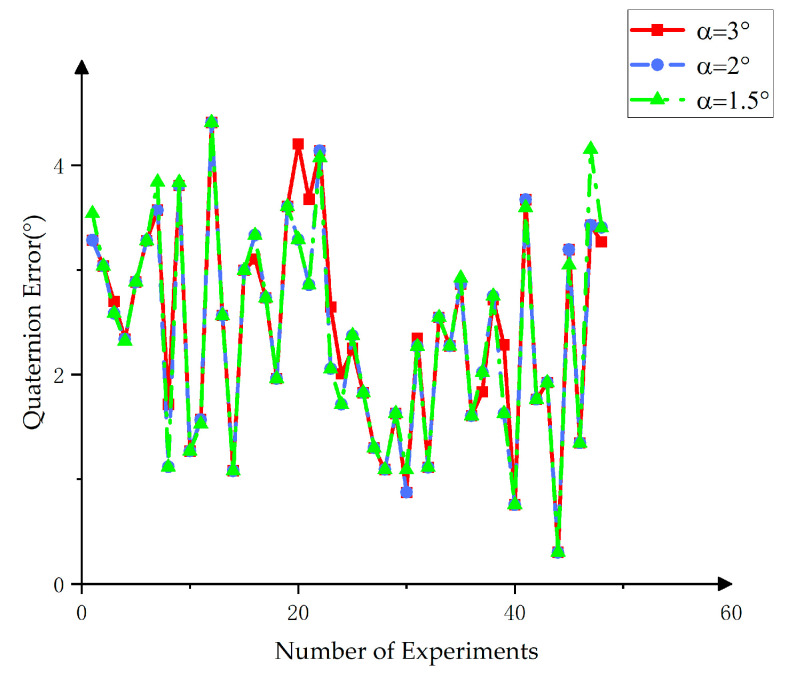
Analysis of experimental results based on different angular thresholds.

A smaller quaternion difference value suggested that the estimated pose was closer to the true pose. As shown in Figure 16, selecting α = 2° as the angular threshold demonstrated the highest accuracy.

**Figure 17:**
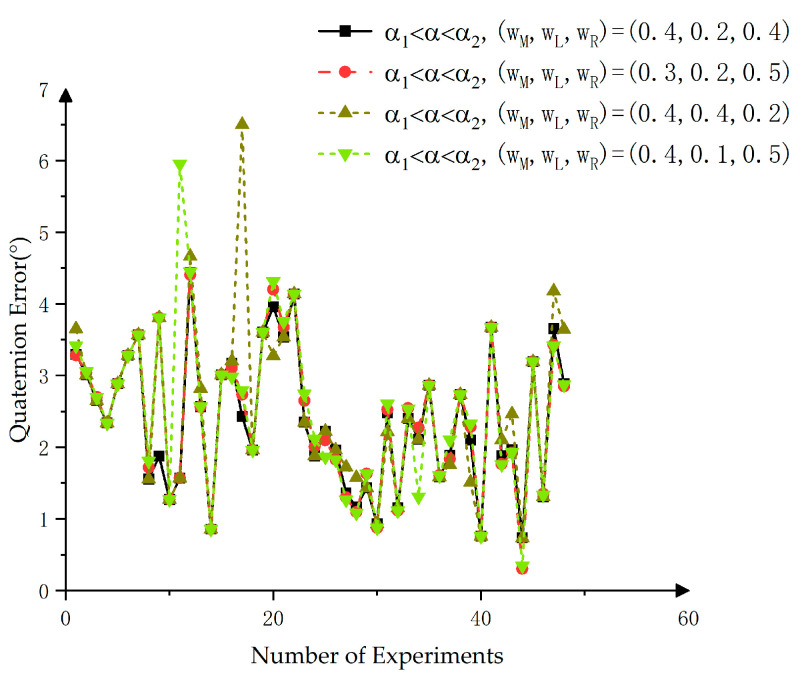
Analysis of experimental results based on initial weight combinations.

After determining the target principal axis angle threshold, the degree of difference between targets can be accurately assessed. Targets with varying degrees of difference in their main bodies were assigned corresponding initial weights $W\left(w_{M},w_{L},w_{R}\right)$. When $\alpha_{1},\alpha_{2}<\alpha$, the weights for the three main bodies of the target were equally distributed as (0.4, 0.3, 0.3), based on the outlined principles. However, when the differences between the target main bodies varied, different weights were assigned according to the specific circumstances. In this paper, four different weighting parameters were tested. Taking the case where $\alpha_{1}<\alpha <\alpha_{2}$ as an example, the weights were set as (0.3, 0.2, 0.5), (0.4, 0.2, 0.4), (0.4, 0.1, 0.5), and (0.4, 0.4, 0.2). In the situation where $\alpha_{2}<\alpha <\alpha_{1}$, the weights were simply reversed. As shown in Figure 17, several overlapping points in the plotted line indicated minimal differences in the target principal axis for these data, suggesting that the choice of weights exerted negligible influence in these cases. Where differences existed, the black line exhibited significantly better performance than the green and brown lines and slightly outperformed the red line. Therefore, the weight distribution of (0.4, 0.2, 0.4) was chosen for cases with uneven differences.

#### 4.3.2. Multi-Target Pose Estimation

**Figure 18:**
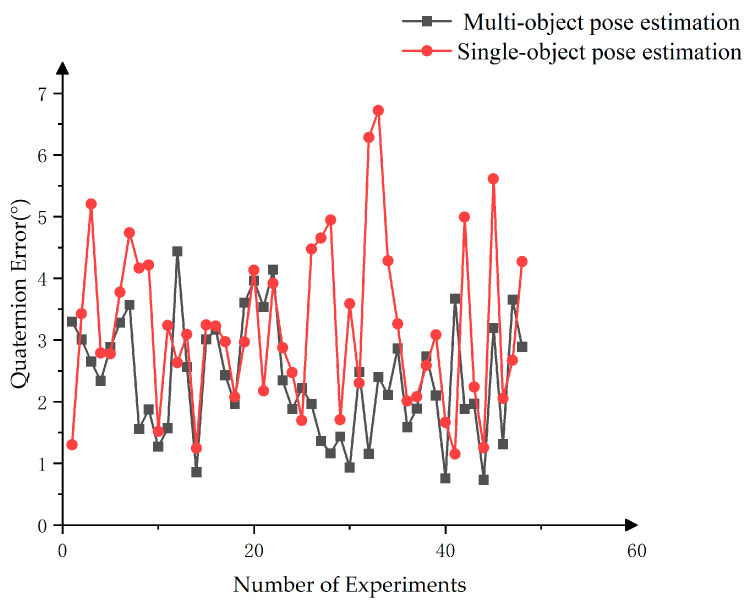
Pose estimation error of single and multi-object subjects.

Figure 18 shows that the multi-target pose estimation method using dynamic weights yielded better results than the approach that relied exclusively on the intermediate target pose as the global pose. As shown in Table 3, the average quaternion difference values for the multi-target pose estimation were consistently lower than those for single-target pose estimation. In terms of variance distribution, the difference values for multi-target pose estimation range from 1 to 4°, while the single-target method exhibited a wider range from 1 to 6.5°, indicating larger extreme values. These results suggest that the multi-target pose estimation method, as employed in this paper, achieved greater stability and a smaller error margin when estimating the poses of actual targets, meeting the practical requirements for robot pose grasping when using only initial weights.

#### 4.3.3. The Impact of Target Principal Axis Pose Confidence on Pose Estimation

**Figure 19:**
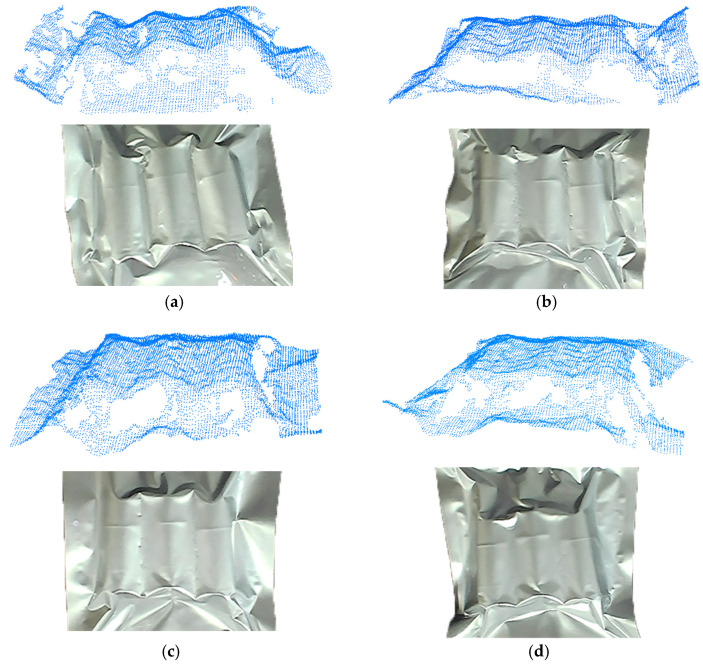
Data types. (**a**) Type A data, characterized by two deep grooves, with the most distinct contour features; (**b**) Type B data, characterized by one deep groove and one shallow groove; (**c**) Type C data, characterized by two shallow grooves; (**d**) Type D data, characterized by the absence of grooves and an extremely indistinct contour, representing defective vacuum-sealed products, which are in an abnormal condition.

To further investigate the impact of edge point cloud loss on object pose estimation, multiple experiments were conducted to evaluate this effect. In addition to the original 48 data sets, 102 new experimental datasets were added. These 102 sets were categorized into three types, A, B, and C, with 34 sets in each category. Figure 19 presents sample images of each type, where Type D corresponds to the abnormal condition.

**Figure 20:**
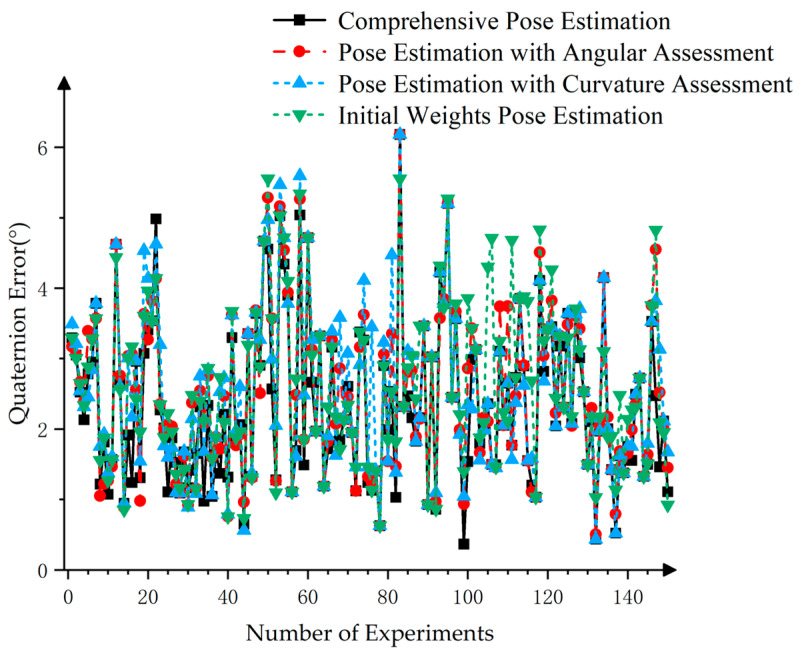
Pose estimation errors using different methods.

As illustrated by the red line in Figure 20, utilizing the difference between the bounding box principal axes and PCA principal axes for target pose assessment, followed by the acquisition of composite pose weight coefficients, results in a marginal enhancement in the final pose estimation. Conversely, the blue line demonstrates that using the concavity and convexity detection of point cloud edges for target pose assessment resulted in unstable weight coefficients for the final pose estimation. The black line demonstrated that a comprehensive score combining both indicators improved the target pose assessment’s weight coefficients for the final pose estimation, with this effect showing a degree of stability due to the assessment of principal axis angle differences.

As demonstrated in Table 4, the error rate of pose estimation using the comprehensive evaluation metrics was reduced to 1.31%. In comparison to the initial weight coefficient pose estimation without any point cloud quality assessment indicators, the enhancement in accuracy, albeit modest, proved effective in further refining the dynamic weight method by employing comprehensive evaluation metrics. More specifically, the dynamic adjustment of the weight coefficient considered the interaction between different quality assessment indicators, such as the principal axis angle difference and the concavity/convexity of point cloud edges. This enabled a more stable and accurate pose estimation, particularly in scenarios where point cloud quality varied. The weight coefficients were adjusted to reflect the relative importance of each indicator, which improved robustness and reduced errors.

#### 4.3.4. Spatial Position Error Analysis

Table 5 presents the error results comparing the calculated center coordinates of the targets with their true values in this study.

**Figure 21:**
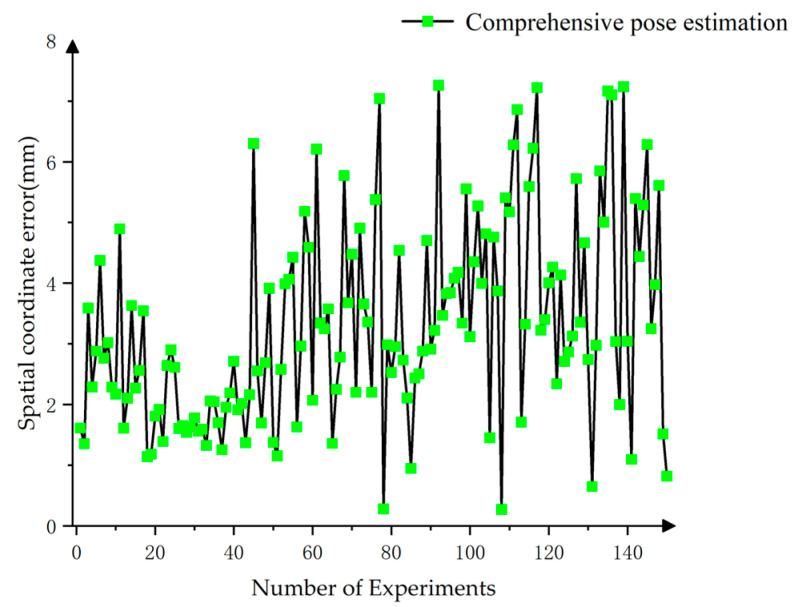
Spatial coordinate error.

The results indicated an overall spatial distance error of 3.278 mm, which was relatively small. Figure 21 presented the line chart of spatial coordinate errors for each data set, showing that the error distribution was primarily within the 1–7 mm range. A substantial portion of the data in the figure showed an error margin of approximately 7 mm, primarily due to variations in the X direction. This was because the central target’s position was more prominent in the X direction compared to the surrounding targets. Specifically, the center of the central target exhibited a significant error in the X direction relative to the overall center of the target ensemble. Despite a few outliers, the errors remained within 1 cm, fulfilling the requirements for robotic target center positioning.

#### 4.3.5. Comparative Experiment

To further demonstrate the superiority of the method proposed in this paper for NMSDP point cloud pose estimation, two classic and widely used pose estimation methods were selected for comparison. The first method was the PPF-based global feature approach for object recognition and pose estimation [23], while the second method was the FPFH-based local feature descriptor approach for point cloud pose estimation. Before applying these methods to the NMSDP point clouds for pose estimation, precise removal of fringe wrinkles was performed on the original NMSDP point clouds to optimize the experimental outcomes. To ensure accurate and robust pose estimations, both methods employed the Iterative Closest Point (ICP) algorithm to refine the pose estimation results. A total of 102 groups of point cloud data, encompassing a variety of types, were selected as the experimental samples.

During NMSDP pose estimation, the target sometimes fails to be accurately recognized or estimated. To facilitate a more comprehensive comparison of different pose estimation methods, this study considers cases with quaternion differences greater than 20° as unsuccessful recognition cases. The metrics in Table 6 were computed based on successfully recognized data. The “Recognition rate” in Table 6 indicates the proportion of successful recognitions. The “Time” metric denotes the runtime of the method. The “Pose error” represented the difference between the estimated pose quaternion and the ground-truth NMSDP pose quaternion. The “Distance error” reflected the spatial distance between the estimated and actual target center coordinates. The results in Table 6 showed that the proposed method achieved superior performance in terms of recognition rate, runtime efficiency, and pose estimation accuracy, demonstrating its effectiveness and robustness in NMSDP pose estimation.

#### 4.3.6. Limitations

To provide a more comprehensive evaluation of the proposed method for NMSDP pose estimation tasks, this study simulated the surface characteristics of Type D samples through manual intervention, generating 228 experimental samples. A target recognition experiment was then conducted on these Type D samples, which occurred with relatively low probability under normal conditions. The results showed 180 successful recognitions and 48 failures, resulting in a recognition rate of 78.95%. The experimental findings indicated that as the surface contours of the NMSDP became less distinct, the recognition efficacy of the proposed method decreased.

## 5. Conclusions

This paper proposed a pose estimation method for NMSDP based on 3D point clouds. The core of this method lies in leveraging surface shape features to segment the target point cloud and combine multi-target and multi-feature information for accurate pose estimation. Specifically, a pre-segmentation method was first employed to eliminate the influence of distorted skirts on the package. An enhanced bounding box orientation filter, applied iteratively, was then used to segment the package and extract the primary target. The final step involved estimating the package’s overall pose using multiple targets and their respective features. The experimental results demonstrated that the multi-target pose estimation approach significantly reduced pose error compared to single-target estimation methods. The accuracy of target pose estimation was further improved by integrating multi-feature evaluations of point cloud quality, yielding an error rate of 1.31%, an average spatial error of 3.278 mm, and an error range of 1–7 mm. Additionally, the proposed method was compared with two classical pose estimation approaches, with experiments showing its superior effectiveness in NMSDP pose estimation tasks. The results suggest that the successful completion of pose estimation tasks may indicate the applicability of the proposed method to other types of molecular sieve drying packages. The limitations of the method were also discussed, with the recognition rate falling short of a high standard when applied to abnormal NMSDP targets.

## Figures and Tables

**Table 1:** Fitting outcomes of bounding boxes at various step sizes.

**Step Sizes/°**	**Time/ms**	**Optimal/Group**	**Success Rate**
0.5	369.739	40	83.33%
1	377.397	43	89.58%
3	431.841	46	95.83%
5	451.053	44	91.67%
7	477.614	44	91.67%
9	502.169	43	89.58%

**Table 2:** Extraction results of the main body point cloud.

**Models**	**Sample Number**	**Accurate Extraction**	**Accuracy Rate**
Unmodified	48	35	73%
Modified	48	48	100%

**Table 3:** Multi-target vs. single-target pose estimation quaternion comparison.

Models	Maximum/mm	Minimum/mm	Mean/mm	Error Rate
Multi-Target	4.43703	0.732062	2.36979	1.31%
Single-Target	6.7208	1.15169	3.27322	1.82%

**Table 4:** Quaternion comparison of pose estimation with various assessment indicators.

Models	Maximum/°	Minimum/deg	Mean/deg	Error Rate
Angular	6.18286	0.50538	2.45180	1.36%
Curvature	6.18286	0.43609	2.50107	1.39%
Initial Weights	5.55494	0.62726	2.54062	1.41%
Comprehensive	6.18286	0.36649	2.36339	1.31%

**Table 5:** Spatial coordinate error.

	Maximum/mm	Minimum/mm	Mean/mm
X Coordinate Error	7.159	0.009	2.469
Y Coordinate Error	6.871	0.003	1.308
Z Coordinate Error	2.920	0.005	0.916
Distance Error	7.260	0.272	3.278

**Table 6:** Comparison of pose estimation methods.

Models	Recognition Rate	Time/s	Pose Error/°	Distance Error/mm
PPF+ICP	50%	27.8	8.584	8.481
SAC-IA+ICP	36.27%	22.6	5.872	8.611
Ours	99.02%	2	2.540	3.771

## Data Availability

Data are not publicly available and can be obtained by contacting the corresponding author if necessary.
